# Amplified fragment length polymorphism of clinical and environmental *Vibrio cholerae *from a freshwater environment in a cholera-endemic area, India

**DOI:** 10.1186/1471-2334-11-249

**Published:** 2011-09-22

**Authors:** Arti Mishra, Neelam Taneja, Ram K Sharma, Rahul Kumar, Naresh C Sharma, Meera Sharma

**Affiliations:** 1 Department of Medical Microbiology. Post Graduate Institute of Medical Education and Research, Chandigarh, 160012, India; 2 Biotechnology Division. Institute of Himalayan Bioresource and Technology, Palampur, Himachal Pradesh, 176061, India; 3 Laboratory Department, Maharishi Valmiki Infectious Diseases Hospital, Kingsway Camp, Delhi 110009, India

## Abstract

**Background:**

The region around Chandigarh in India has witnessed a resurgence of cholera. However, isolation of *V. cholerae *O1 from the environment is infrequent. Therefore, to study whether environmental nonO1-nonO139 isolates, which are native to the aquatic ecosystem, act as precursors for pathogenic O1 strains, their virulence potential and evolutionary relatedness was checked.

**Methods:**

*V. cholerae *was isolated from clinical cases of cholera and from water and plankton samples collected from freshwater bodies and cholera-affected areas. PCR analysis for the *ctxA, ctxB, tcpA, toxT *and *toxR *genes and AFLP with six primer combinations was performed on 52 isolates (13 clinical, 34 environmental and 5 reference strains).

**Results:**

All clinical and 3 environmental isolates belonged to serogroup O1 and remaining 31 environmental *V. cholerae *were nonO1-nonO139. Serogroup O1 isolates were *ctxA, tcpA *(ElTor), *ctxB *(Classical), *toxR *and *toxT *positive. NonO1-nonO139 isolates possessed *toxR*, but lacked *ctxA *and *ctxB*; only one isolate was positive for *toxT *and *tcpA*. Using AFLP, 2.08% of the *V. cholerae *genome was interrogated. Dendrogram analysis showed one large heterogeneous clade (n = 41), with two compact and distinct subclades (1a and 1b), and six small mono-phyletic groups. Although *V. cholerae *O1 isolates formed a distinct compact subclade, they were not clonal. A clinical O1 strain clustered with the nonO1-nonO139 isolates; one strain exhibited 70% similarity to the Classical control strain, and all O1 strains possessed an ElTor variant-specific fragment identified with primer ECMT. Few nonO1-nonO139 isolates from widely separated geographical locations intermingled together. Three environmental O1 isolates exhibited similar profiles to clinical O1 isolates.

**Conclusion:**

In a unique study from freshwater environs of a cholera-endemic area in India over a narrow time frame, environmental *V. cholerae *population was found to be highly heterogeneous, diverse and devoid of major virulence genes. O1 and nonO1-nonO139 isolates showed distinct lineages. Clinical isolates were not clonal but were closely related, indicating accumulation of genetic differences over a short time span. Though, environment plays an important role in the spread of cholera, the possibility of an origin of pathogenic O1 strains from environmental nonO1-nonO139 strains seems to be remote in our region.

## Background

Cholera continues to be a serious epidemic disease in various parts of Asia and Africa [1]. It is a severe form of acute secretory diarrhoea caused by a gamma proteobacterium, *Vibrio cholerae *[2]. More than 200 serogroups (O1-O200) exist for *V. cholerae *on the basis of epitopic variations in cell surface lipopolysaccharides, but only serogroups O1 and O139 are pathogenic, and are associated with cholera [3]. The pathogenic *V. cholerae *relies on the synergistic action of a set of virulence genes for pathogenesis in humans. These include the CTX element [4] and vibrio pathogenicity island (VPI) [5], which encode cholera toxin (CT) and a colonization factor, toxin-coregulated pilus (TCP) respectively. Other important genes include *toxR*; this encodes a master regulatory protein which, along with another factor encoded by *toxT*, coregulates expression of both CT and TCP [6]. *V. cholerae *is prone to extensive horizontal gene acquisition of CTX and VPI elements [7]. Consequently, conversion of non-toxigenic strains into pathogenic strains is possible by horizontal acquisition of virulence genes. It has been reported that the transduction process in the environment can result in conversion of non-toxigenic environmental strains into toxigenic strains [8].

The aquatic ecosystem has been incriminated for a long time as a source and reservoir for *V. cholerae *[9]. There are several published reports regarding the distribution of virulence genes in environmental strains of *V. cholerae*, which support the possibility of an environmental origin of pathogenic *V. cholerae *[10,11]. The Northern region of India, which has freshwater environments and is far away from the sea, has witnessed a resurgence of cholera in the recent past. In addition to frequent sporadic cases, seasonal outbreaks have occurred around Chandigarh in 2002 [12], 2004 [13], 2007 [14] and 2008 [15]. In the present study, the distribution of virulence genes and the molecular relatedness of environmental isolates of *V. cholerae *to clinical isolates from patients with cholera from Chandigarh and the surrounding region were investigated. Amplified fragment length polymorphism (AFLP) was applied to investigate the evolutionary relationships between environmental *V. cholerae *and clinical isolates in order to understand the origin, epidemiology and spread of cholera in an endemic area with freshwater environs.

## Methods

### Collection and processing of samples

The present study was conducted around Chandigarh, a region in North India (Latitude: 30°43' N, Longitude: 76°47' E). Two groups of samples for the isolation of *V. cholerae *were collected: environmental and clinical samples. The clinical *V. cholerae *were isolated from stool samples of patients infected with cholera that were submitted to the Enteric Laboratory, Department of Medical Microbiology, PGIMER, Chandigarh, India. This is an 1800-bed tertiary care referral institute in North India that caters to a vast population in five states: Punjab, Haryana, Jammu and Kashmir, Himachal Pradesh and Uttrakhand. The environmental samples (water and plankton) were collected from freshwater bodies and cholera-affected areas around Chandigarh (Additional files 1 and 2). Briefly, water samples were collected in sterilized narrow mouthed bottles, and 1.0 L was filtered through 0.22- μm pore-sized nitrocellulose acetate filter membranes (Millipore, Bethesda, USA) which were washed with phosphate buffered saline (PBS, pH 7.4). Plankton samples were collected by filtering 20.0 L of water through a plankton net (mesh size 25.0 μm) disinfected previously with 70% ethyl alcohol. The plankton samples were washed with PBS and concentrated in sterile plastic tubes. Aliquots (1.0 ml) of sample concentrate from water and plankton sampleswere added to doubly concentrated alkaline peptone water (APW). After incubation for 6 h at 37°C, a loopful of incubated APW was streaked onto selective thiosulphate citrate bile salt sucrose agar medium (Difco, Detroit, MI, USA) and incubated for an additional 24-h period at 37°C. Single colonies typical of *V. cholerae *i.e. yellow (sucrose fermenting) and flat (2 to 3 mm in diameter) were transferred to 10% sheep blood agar. The environmental *V. cholerae *isolates were characterized extensively by a battery of biochemical reactions including oxidase, Hugh and Leifson O/F test, Moeller's arginine dihydrolase, lysine and ornithine decarboxylases, arabinose, mannose, sucrose peptone water sugars, the string test and cholera red reaction [16]. After phenotypic characterization, the isolates were confirmed genotypically as *V. cholerae *by species-specific PCR for the *ompW *gene [17]. The isolates were further tested serologically with commercially available *V. cholerae *O1 and O139 antiserum (Denka Seiken, Japan Ltd.).

### PCR assays for virulence genes

The *V. cholerae *isolates were subjected to PCR assays, described previously, for various virulence genes. The targeted DNA sequences were: *ctxA *and *tcpA *[18], *ctxB *[19], *toxR *and *toxT *[20]. The oligonucleotides were synthesized by Invitrogen, India. The genomic DNA was extracted using a protocol described previously [21]. The PCR reaction was carried out in a final volume of 25 μl containing: 20 mM Tris-HCl (pH 8.3), 50 mM KCl, 1.5 mM MgCl_2_, 0.001% gelatin, 100 μM of each dNTP, 0.8 μM primer (20 pmol/reaction), 1U Taq DNA polymerase and 25-100 ng of template DNA (all reagents from Banglore Genei, India). The following standard strains were used for the phenotypic and genotypic tests: *V. cholerae *O1 ElTor N16961 (ElTor), *V. cholerae *O1 Classical 569B (Classical), *V. cholerae *O1 ElTor variant (ElTor variant), *V. cholerae *nonO1-nonO139 NT5394 (NT5394); all were obtained from the National Institute of Cholera and Enteric Diseases (NICED), Kolkata, India. In addition, *V. cholerae *O1 MTCC 3906 ElTor (MTCC 3906) was obtained from the Microbial Type Culture Collection (MTCC) of the Institute of Microbial Technology, Chandigarh, India.

### Amplified fragment length polymorphism analysis

Genotyping of *V. cholerae *was performed by AFLP as described previously [22]. In brief, 125.0 ng of DNA was double digested with *Eco*RI/*Mse*I (1.25 U/μl) at 37°C for 2 h. The restriction endonucleases were then inactivated at 70°C for 15 min and subsequent ligation of adapters was performed at 20°C for 2 h. The sequences of primers and adapters used are listed in Additional file 3. The restricted ligated mixture was diluted 1:5 and subjected to a preamplification reaction using non selective primers (E-0, M-0; with no additional selective base) to amplify all *Eco*RI-*Mse*I fragments. Preamplification was done with a PCR mix containing: 3.5 μl DNA, 1.5 μl each EcoRI/MseI primer (30 ng), 0.1 μl 200 μM dNTPs, 0.33 μl Taq polymerase (5U/μl), 2.5 μl 10 × PCR buffer containing 15 mM MgCl_2_, 1.0 μl MgCl_2 _(10 mM) and 14.67 μl H_2_O. The cycling profile used was: 20 cycles of denaturation at 94°C for 30 s, annealing at 56°C for 60 s and extension at 72°C for 60 s. After preamplification the reaction mixture was diluted 1:50 in TE buffer (pH 8.0) and subjected to selective amplification using selective primers G/G, C/T, C/G, G/T, T/A and G/A (E-N, M-N; with +1additional base). The selective amplification was carried out using 1.0 μl template (25 ng/μl of preamplicon), 1.5 μl each EcoRI/MseI primer (30.0 ng), 0.1 μl 200 μM dNTPs, 0.17 μl Taq polymerase (5 U/μl), 1.0 μl 10 × PCR buffer containing 15.0 mM MgCl_2 _and 4.73 μl H_2_O. The touchdown cycling profile for selective amplification was as follows: cycle 1: 94°C for 30 s, 65°C for 30 s, 72°C for 60 s; cycles 2 to 13: similar to cycle 1 except for a stepwise decrease in the annealing temperature in each cycle by 0.7°C; cycles 14 to 36: 94°C for 30 s, 56°C for 30 s, 72°C for 60 s. Following selective amplification, the reaction products were separated on 6% denaturing polyacrylamide gels using SequiGen gel apparatus 38 × 50 × 0.4 cm (BioRad Laboratories Inc., Hercules, CA, USA) and developed by silver staining (Promega; WI, USA).

### Evaluation of AFLP data

Bands from the AFLP gels were scored manually, as '1' if present and '0' when absent. The binary data were evaluated according to the Jaccard coefficient of similarity and a phylogenetic tree (dendrogram) was constructed using the unweighted pair group method with arithmetic mean (UPGMA) [23] following sequential agglomerative hierarchical nested (SAHN) cluster analysis using NTYsysPC version 2.003e (Applied Biostatics Inc.). The discriminatory power was calculated using the Simpson's coefficient of diversity (D) by using the formula D = 1 - {Σ[nj(nj - 1)]}/[N(N - 1)], where N is the number of strains tested, and nj denotes the number of strains belonging to the j^th ^type [24]. Principal coordinate analysis (PCoA) was performed using DARwin 5.0 [25,26]. To investigate the variability within the entire *V. cholerae *population further, principal components analysis (PCA) [27] was also performed using informative fragments with the Software Package for Statistics and Stimulation (SPSS 16.0), employing Varimax with Kaiser normalization (rotation method). The chi-square and Fisher exact probability tests were used to test the significance of the presence of fragments in clinical isolates. The percentage of the genome interrogated using AFLP was calculated as described previously by Lan and Reeves [28]. The average length of the *Eco*RI-*Mse*I AFLP fragment is 256 bp (equivalent to the expected frequency of tetracutter *Mse*I) and 9 nucleotides are associated with each *Eco*RI-*Mse*I fragment (3 bases for the *Eco*RI site; 4 for the *Mse*I site, and 2 for +1 selection at each primer). Therefore, the genome studied for internal length variation is equivalent to the number of fragments scored multiplied by 256 bp, and point mutations scored are calculated by multiplying the number of fragments by 9 bp.

## Results

### Characterization of *V. cholerae *isolates

Fifty-two *V. cholerae *isolates (13 clinical isolates, 34 environmental isolates and 5 reference strains) were included in the present study. All clinical and three environmental isolates belonged to serogroup O1 and the remaining 31 environmental *V. cholerae *were nonO1-nonO139. The *V. cholerae *O1 were positive for *ctxA, tcpA *(Eltor), *ctxB *(Classical), *toxR *and *toxT*. All nonO1-nonO139 isolates were positive for *toxR *and negative for *ctxA *and *ctx B*. All except one nonO1-nonO139 isolate (E34/FS) were negative for *tcpA *(ElTor) and *toxT*.

### Genotyping

A total of 318 fragments were scored, ranging in size from 75 to 700 bp (Table 1). Two hundred and ninety-nine fragments (94.3%) were polymorphic and nineteen fragments (5.6%) were monomorphic. The primer combination G/T detected the largest number of polymorphic bands (68), while the primer pair G/G detected the fewest (26) polymorphic fragments, with an average of 50 polymorphic fragments per primer pair. Two hundred and eighty-two fragments were phylogenetically informative (88.9%); out of these the majority (97.51%) were found to be overlapping between the environmental and the clinical strains. No group-specific fragments, exclusive to either environmental or clinical isolates, were found. However, seven fragments were obtained that were predominant in O1 strains as compared to nonO1-nonO139 strains (p < 0.001). One fragment (240 bp) obtained with ECMT primer combination specific for *V. cholerae *ElTor variant (Kolkata, NICED) was present in all O1 isolates but absent in other reference strains. The discriminatory value (D) for each of the six primer pairs used was greater than 0.98 (Table 1). Overall, using AFLP, 2.08% of the 4,034,664-bp *V. cholerae ge*nome [29] was interrogated, including 81,408 nucleotides for *Eco*RI-*Mse*I internal length variation (318 fragments × 256 bp) and nearly 2,862 for point mutations (318 fragments × 9 bp).

**Table 1:** Fragments obtained during AFLP analysis using six primer combinations in *V. cholerae*.

Pair No	Primers^a^	No. of fragments analyzed					D^g^
			
		Total bands^b^	Monomorphic^c^	Polymorphic^d^	Informative^e^	Singular^f^	
1.	G/G	29	3	26	25	1	0.99

2.	C/T	68	3	65	59	6	0.98

3.	C/G	64	6	58	54	4	0.99

4.	G/T	69	1	68	68	0	0.99

5.	T/A	43	1	42	38	4	0.98

6.	G/A	45	5	40	38	2	0.99

Total		318	19	299	282	17	0.99

^a ^Denoting *Eco*RI and *Mse*I primers, each with one selective base (*Eco*RI-*Mse*I +1/+1). The letters such as G/G, indicate selective bases respectively for *Eco*RI and *Mse*I.

^b ^Bands scored excluding some small and large fragments at the ends of the gel.

^c ^Monomorphic refers to fragments present in all isolates.

^d ^Fragments showing variation in their patterns among all isolates.

^e ^Fragments present or absent in two or more isolates.

^f ^Fragment present or absent only in one isolate.

^g ^D-Simpson's index of diversity.

### Diversity and clonality of *V. cholerae *based on genotyping

**Figure 1:**
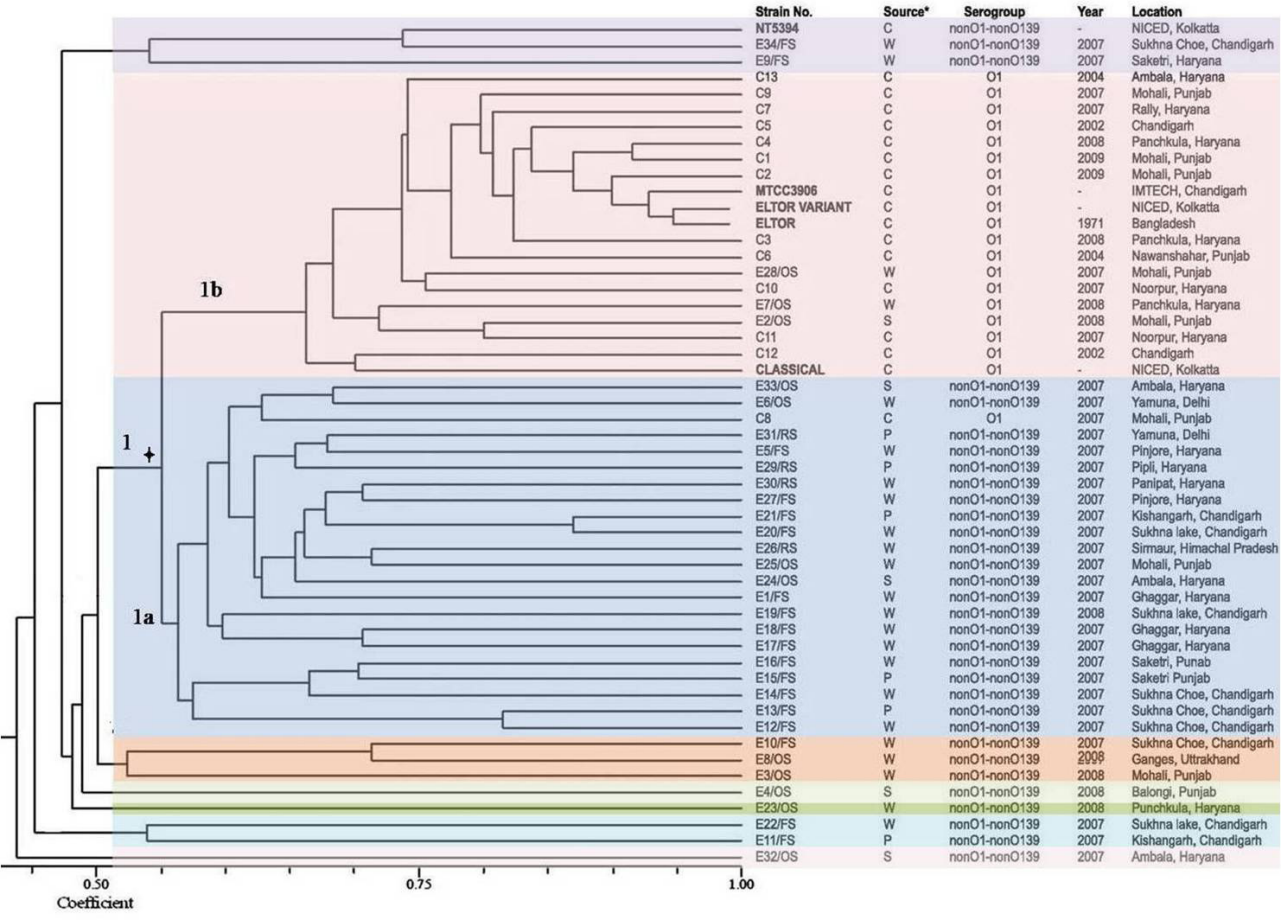
**Dendrogram of 52 *V. cholerae *isolates after *EcoRI/MseI *restriction digestion and amplification with six primer combinations**. The dendrogram was constructed using UPGMA algorithm based on Jaccard Coefficient obtained after pairwise comparison of AFLP variation. *Source: W- water P-plankton S-sewage C-Clinical isolates.

The AFLP analysis of the isolates demonstrated clearly that the *V. cholerae *isolates in this cholera-endemic region form a highly diverse population. By dendrogram isolates examined were divided into one large heterogeneous clade (n = 41), with the two compact and distinct subclades (1a and 1b), and six small mono-phyletic groups with one to three members in each (Figure 1). The distribution of isolates did not correlate with a particular time frame or geographical location. Sub-clade 1a (n = 22) consisted of nontoxigenic environmental *V. cholerae *nonO1-nonO139 isolated from water and plankton samples from different locations (mean similarity index 0.56). Only one pathogenic clinical O1 strain, C8 from 2007 (C8/07), belonged to sub-clade 1a. Sub-clade 1b (n = 19) consisted of clinical isolates from patients in North India collected between 2002 and 2009 and environmental toxigenic O1 strains: the O1 isolates collected at the time of an outbreak in 2008 (mean similarity index 0.63) and all the O1 reference strains. Interestingly the ElTor and ElTor variant type strains showed almost 94% pattern similarity. All O1 isolates exhibited 66% similarity with the Classical type strain. In this cluster a conspicuous subgroup comprised of a clinical isolate (C12) isolated in 2002 and the Classical type strain was observed (similarity index 0.70). In addition to the above-mentioned subclades, six monophyletic groups - consisting of three singletons (E4/OS, E23/OS, E32/OS), and three small groups consisting of 2 to 3 members were observed. All of them were separated from the main clade with a similarity index less than 0.54. NT5394, a clinical nonO1-nonO139 isolated in Kolkata, E34/FS (the only environmental nonO1-nonO139 isolate that was *tcpA*+ and *toxT*+) and E9/FS, isolated from two separate locations around Chandigarh in 2007, clustered together. Overall, toxigenic *V. cholerae *O1 strains from the environment and clinical samples clustered together, while nonO1-nonO139 strains diverged widely from the O1 isolates.

**Figure 2:**
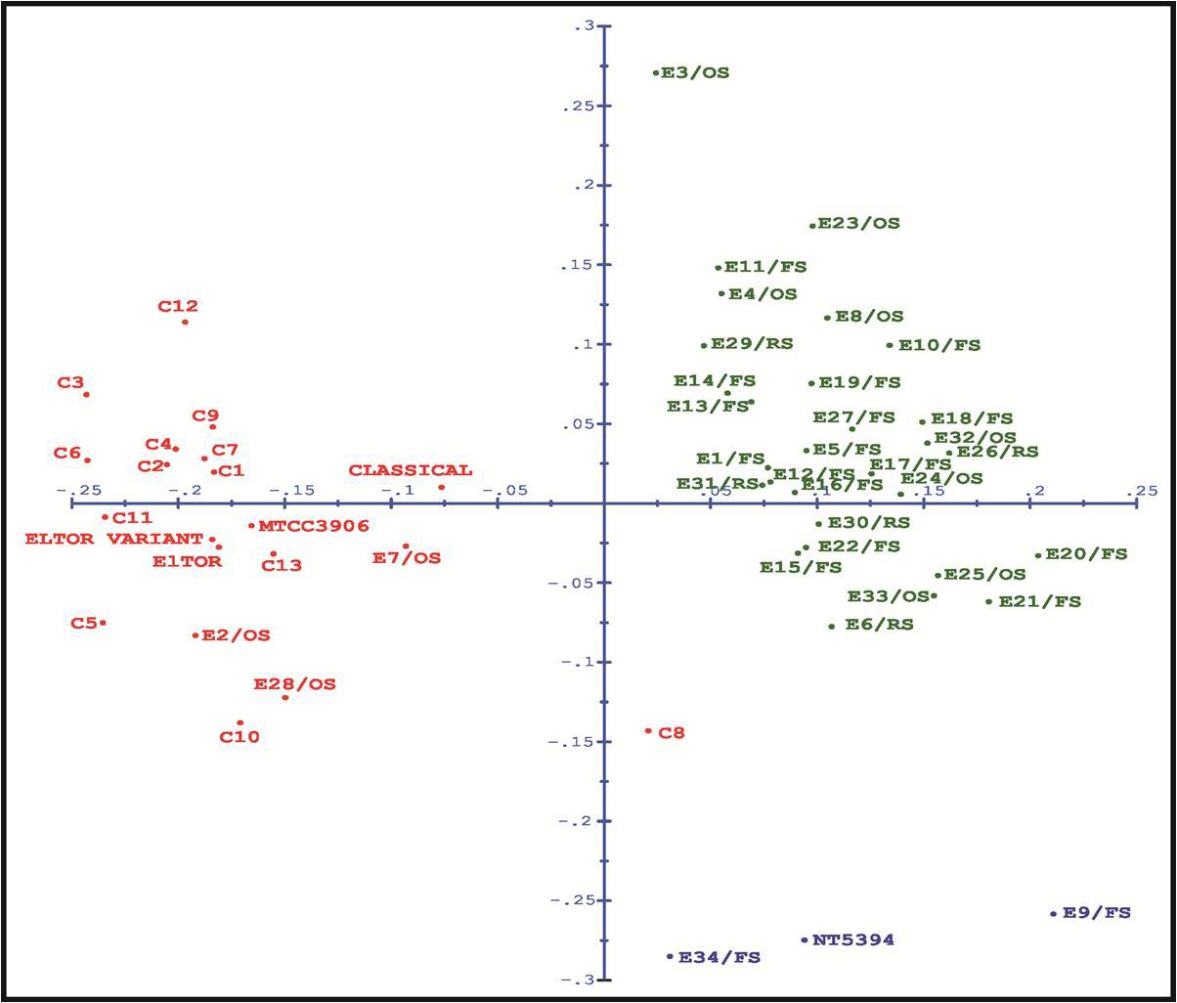
**Principal coordinate analysis (PCoA) plot for *V. cholerae *population based on AFLP fingerprinting pattern**. The isolates are represented as points in the ordination space. O1 isolates are depicted in red and nonO1-nonO139 in green and blue.

**Figure 3:**
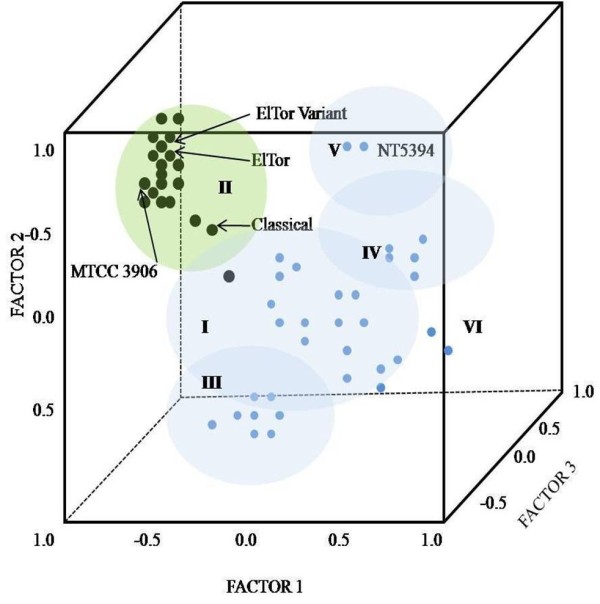
**Three dimensional representation of *V. cholerae *population from cholera endemic area using Principal component analysis (PCA) derived from informative fragments**. Black dots indicate O1 strains while blue dots indicate nonO1-nonO139 strains. I, II, III, IV, V and VI represents 6 components into which 52 isolates were divided. All *V. cholerae *O1 belonged to component II except C8/07 belonging to component I and nonO1-nonO139 isolates exhibited heterogeneous distribution.

Using dendrogram analysis of the individual primer combinations C/T, G/T and G/A, similar observations were made (data not shown) i.e. all O1 isolates clustered together and nonO1-nonO139 were very heterogeneous. However, with the remaining primer combinations, G/G, C/G and T/A, a larger number of singletons was observed for both clinical and environmental isolates. Using principal co-ordinate analysis, all the isolates were divided into two groups (pathogenic O1 and nonpathogenic nonO1-nonO139 isolates) along the second coordinate axis, except for C8/07 which is an O1 isolate but was grouped with nonO1-nonO139 (Figure 2). Overall, the distribution for nonO1-nonO139 isolates was quite heterogeneous. The principal component analysis (Figure 3), using informative fragments (282), reduced the 52 *V. cholerae *into six components (18, 17, 8, 5, 2 and 2 isolates in each component). In three-dimensional space, all *V. cholerae *O1, including the reference strains, clustered together in component II except C8/07. Environmental nonO1-nonO139 isolates again constituted a heterogeneous population (components I, III, IV, V and VI), further supporting the dendrogram analysis.

## Discussion

This study was carried out in a cholera-endemic region around Chandigarh that has freshwater environs. This region has witnessed recently a resurgence of cholera, with frequent outbreaks during summers and rainy seasons associated with contaminated water. However, isolation of *V. cholerae *O1 in the laboratory is infrequent. Therefore, to study whether environmental nonO1-nonO139 isolates, which are native to the aquatic ecosystem, act as precursors for pathogenic O1 strains, their virulence potential and evolutionary relatedness was checked. AFLP analysis was performed with six primer combinations on 52 *V. cholerae *isolates for realistic exploration of genome composition, which facilitated strain-to-strain discrimination. We investigated the relationships between clinical and environmental isolates on the basis of random interrogation of nearly 2.08% of the genome. The isolates, irrespective of their origin, were divided mainly into a large single heterogeneous clade, and six mono-phyletic groups. All the O1 isolates from cholera patients and the environment harboured the major virulence factors *ctxA *and *tcpA *and belonged to a single sub-clade (1b). The environmental nonO1-nonO139 isolates did not possess major virulence genes and were heterogeneous in their distribution as shown by the dendrogram, PCoA and PCA analyses. Overall the resolving power for AFLP was found to be very high (0.99), as documented previously [28].

For the clinical isolates from different outbreaks, the dendrogram did not show a distinct clonal population (similarity index < 95%). Similarly, the *V. cholerae *O1 isolates formed a loose group in PCoA. This denotes that continuous evolution is occurring in the *V. cholerae *O1 population, which could be due to its ability for extensive horizontal gene exchange. However, through PCA, using only informative fragments and excluding the singular fragments, there was evidence of clonal proliferation in the form of a tight cluster. Therefore, we concluded that clinical isolates of *V. cholerae *obtained in the same year were closely related. These strains may give rise to outbreaks as a result of rapid expansion at particular time intervals, and must be accumulating genetic differences over time. Interestingly, fingerprint patterns similar to those of clinical strains were exhibited by three environmental O1 strains. These were isolated during outbreaks from natural waters in the cholera affected area, demonstrating that the aquatic environment can serve as a reservoir for the transmission and spread of cholera, as already established by epidemiological studies of cholera [30].

This study revealed some interesting findings. One clinical strain of *V. cholerae *O1 Ogawa ElTor (C12), isolated in 2002, clustered with the Classical type strain (mean similarity index 0.70 as compared to 0.66 for others). *V. cholerae *O1 Classical has not been isolated from this region since 1975 (unpublished data). The control ElTor variant from NICED Kolkata exhibited approximately 94.0% similarity with Eltor N16961. In addition, seven fragments were found to be predominant in O1 strains as compared to nonO1-nonO139 strains. Surprisingly, we found an ElTor variant-specific fragment that was absent from ElTor N16961, Classical and MTCC 3906 type strains and was present in all O1 isolates. The presence of this specific fragment signifies that ElTor variants are circulating in our environment. Currently these variants are the predominant clone circulating worldwide [31]. We confirmed our findings by detecting the presence of *ctxB *(Classical) by mismatch amplification mutation assay (MAMA-PCR). A clinical isolate of *V. cholerae *O1 (C8/07), isolated from the 2007 outbreak, clustered with nontoxigenic environmental isolates in sub-clade 1a that were isolated from different geographical locations(0.64 similarity index with E33/OS and E6/OS). The significance of this finding is not clear, but it may imply that some clinical isolates have originated from environmental strains or from a common ancestor.

The environmental nonO1-nonO139 isolates exhibited very high divergence in their patterns. A strain isolated from Ganges, and two strains isolated from Yamuna, which are two major rivers in India, clustered with isolates from natural waters in widely separated geographical regions in Punjab and Haryana. In addition, two strains isolated from natural waters in North India clustered with NT5394, a clinical nonO1-nonO139 strain isolated in NICED, Kolkata. Therefore, the presence of some strains with related genomes in completely separate geographical locations suggests that *V. cholerae *is highly successful in adapting to changing environmental conditions, as reported previously [32]. Apart from the above findings, *V. cholerae *isolated from different geographical locations and time periods intermingled in sub-clade 1a. The O1 and nonO1-nonO139 isolates were distinguishable clearly in the dendrogram, and in PCoA and PCA analyses. Therefore, the pathogenic *V. cholerae *O1 were genetically different and might have evolved from distinct lineages.

The extensive variation in AFLP patterns in *V. cholerae *from a small geographical area leads us to conclude that this organism is very diverse and is evolving continuously. Zo *et al*. in Bangladesh studied the spatial and temporal relationship between clinical and environmental isolates from the same geographical areas by enterobacterial repetitive intergenic consensus (ERIC) PCR and found geographical seclusion to be a predominant factor in the creation of different lineages of *V. cholerae *[33]. In our study, *V. cholerae *strains were studied over a much narrower time frame because almost 30 isolates were obtained in 2007. The large number of DNA polymorphisms studied in this short time frame revealed that the entire population of *V. cholerae *in this region is highly heterogeneous and diverse, and that strains from different geographic regions are intermingled, implying a weak spatial relation. This difference could also be due to the high discriminatory power of AFLP versus ERIC-PCR. Similar to our study, extensive variation in a short time frame and small geographical location has been observed using variable number of tandem repeat (VNTR) analysis in Bangladesh [34]. The limited overlap between clinical and environmental isolates in the present study is in concordance with the above-mentioned study. Similarly, the present study does not support the concept of seasonal cholera outbreaks occurring by movement of a single clonal wave across the region, because clinical isolates from the same years were not clonal. *V. cholerae *O1 may cause outbreaks by rapid expansion at particular time intervals, and must be accumulating genetic differences over time. Comparative genome sequencing will provide the most definitive answer to this question. In a recent study by Chun *et al*. using genome based phylogeny, it was concluded that *V. cholerae *O1 undergoes extensive genetic recombination via lateral gene transfer driven by environmental factors. The pandemic clones are drifting as a result of variations in the composition of laterally transferred genomic islands, which results in *V. cholerae *O1 Eltor hybrid/variant clones [35]. Our study also supports the above hypothesis because the ElTor and ElTor variant type strains showed 94% similarity. In the above mentioned study, phylogenetic analysis revealed that the strains belonging to nonO1-nonO139 serogroups from various sources showed significant genomic diversity. Therefore, taking into consideration the abundance of vibrios (0.5 to 4% of aquatic bacteria) in the environment [36], and bearing in mind the extensive genetic variation that these organisms undergo, the *V. cholerae *in this cholera-endemic area were concluded to be evolving ad infinitum. The possibility of the origin of pathogenic strains from environmental strains seems to be limited, because the O1 and nonO1-nonO139 isolates were overall very diverse in our region.

## Conclusions

This study is unique because it was carried out in a cholera-endemic region and over a narrow time frame. It is the first such study from an area of India with a freshwater environment. In this study, we investigated the relationship between clinical and environmental isolates of *V. cholerae *on the basis of random interrogation of 2.08% of the genome by AFLP, a highly discriminatory technique, using six primer combinations. The nonO1-nonO139 strains were clearly distinguishable from pathogenic O1 stains because they did not possess any major virulence genes on PCR analysis and exhibited different AFLP patterns. The *V. cholerae *O1 population was not clonal but was closely related. Our study does not support the concept of seasonal cholera outbreaks that occur by movement of a single clonal wave across the region, because the clinical isolates from the same years were clearly different. This signifies that continuous evolution is occurring in *V. cholerae *strains. *V. cholerae *O1 isolates from patients with cholera and from the aquatic environment belonged to a single cluster, which demonstrated the role of the aquatic ecosystem in the spread of cholera. The nonO1-nonO139 strains were very heterogeneous in their patterns and were different genetically from the O1 strains. The precise role of nonO1-nonO139 strains in the dynamics of cholera outbreaks is unknown at present. Our results suggest that they are highly diverse and may be contributing to diversity in this cholera endemic area by extensive genetic recombination via horizontal gene transfer. The possibility of an origin of pathogenic O1 strains from nonO1-nonO139 environmental strains does not seem to be likely in our region, because the nonO1-nonO139 isolates were nonpathogenic overall and diverse, and only one clinical isolate clustered with the environmental nonO1-nonO139 *V. cholerae *strains.

## Competing interests

The authors declare that they have no competing interests.

## Authors' contributions

NT, AM and MS designed the study. AM performed collection and processing of samples under the supervision of NT. NCS helped and provided laboratory support for sample collection from their region. AM and RK performed AFLP under the guidance of RKS. AM and NT wrote the paper. All authors have read and approved the final manuscript.

## Pre-publication history

The pre-publication history for this paper can be accessed here:

http://www.biomedcentral.com/1471-2334/11/249/prepub

## Supplementary Material

Additional file 1 **Figure showing sites of fixed sample collection**. Eight fixed water sample collection sites were: Jayanti Devi, Saketrii, Sukhna Choe, Sukhna Lake, Pinjore, Ghaggar river at Nadda Sahib, Kishangarh, and Derrabasi. Samples were collected in between April 2007-March 2008 and *V. cholerae *were isolated. Click here for file

Additional file 2 **Figure showing region of collection of clinical samples and environmental samples from random natural water sites in north India**. The clinical *V. cholerae *O1 included in this study were collected from - Mohali, Nawanshar (Punjab), Rally (near Panchkula), Panchkula, Ambala and Noorpur (Haryana). The samples from rivers of North India *viz*. Satluj, Yamuna, and Ganges were collected at Bhakra (Ropar, Punjab), Delhi and Haridwar (Uttrakhand) respectively. The freshwater samples were also collected from Sirmaur (Himachal Pradesh) and Pipli (Haryana). Click here for file

Additional file 3 **Table showing sequence of primers and adapters**. Sequences of primers and adapters used in this study. Click here for file
