# Supplementary figures and images for: Amplified fragment length polymorphism of clinical and environmental Vibrio cholerae from a freshwater environment in a cholera-endemic area, India
Source: BMC Infect Dis. 2011 Sep 22;11:249. doi: 10.1186/1471-2334-11-249 (PMC3206463; doi:10.1186/1471-2334-11-249)

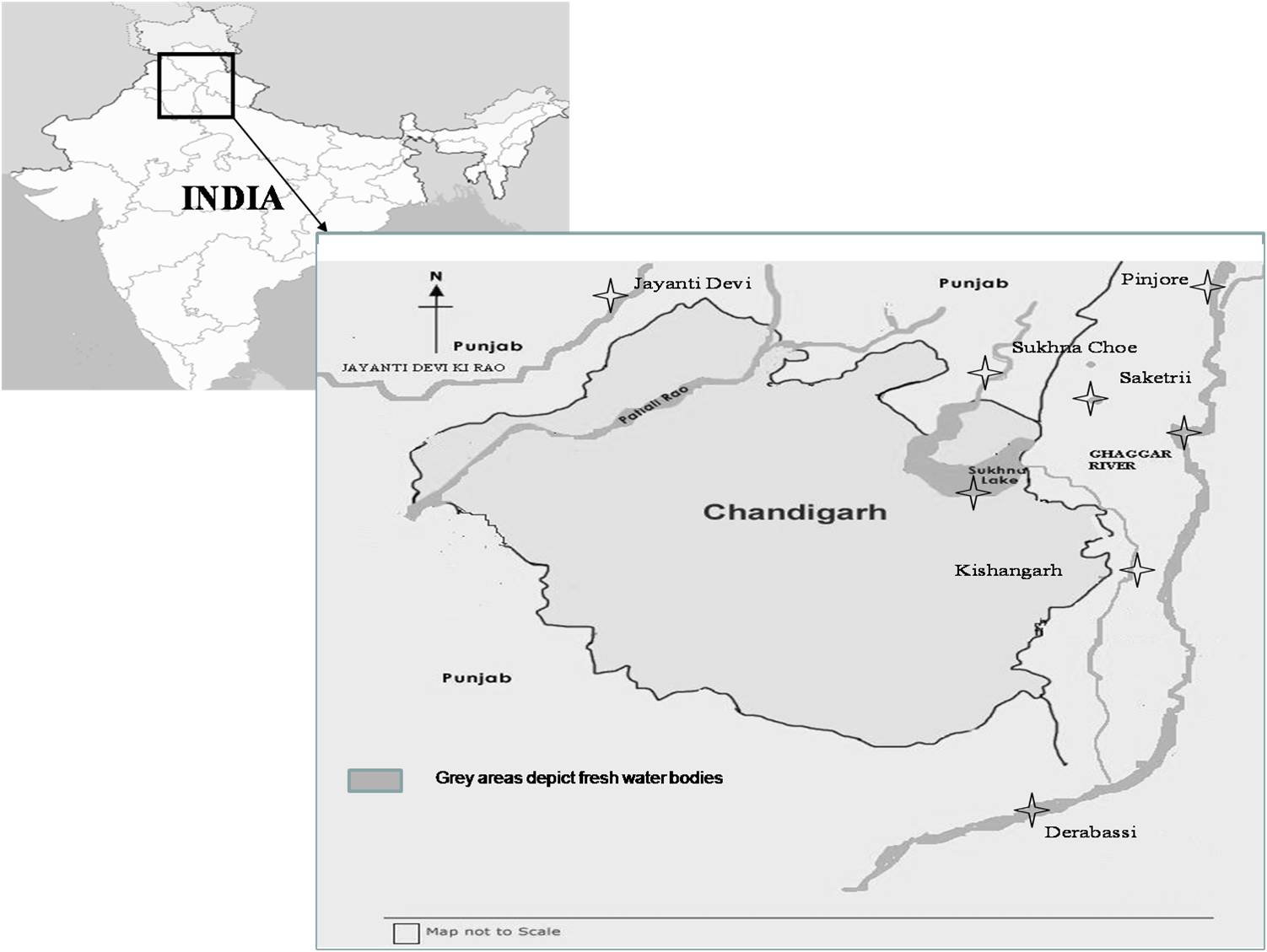

Supplement: Additional file 1 — Figure showing sites of fixed sample collection. Eight fixed water sample collection sites were: Jayanti Devi, Saketrii, Sukhna Choe, Sukhna Lake, Pinjore, Ghaggar river at Nadda Sahib, Kishangarh, and Derrabasi. Samples were collected in between April 2007-March 2008 and V. cholerae were isolated. [file 1471-2334-11-249-S1.JPEG]

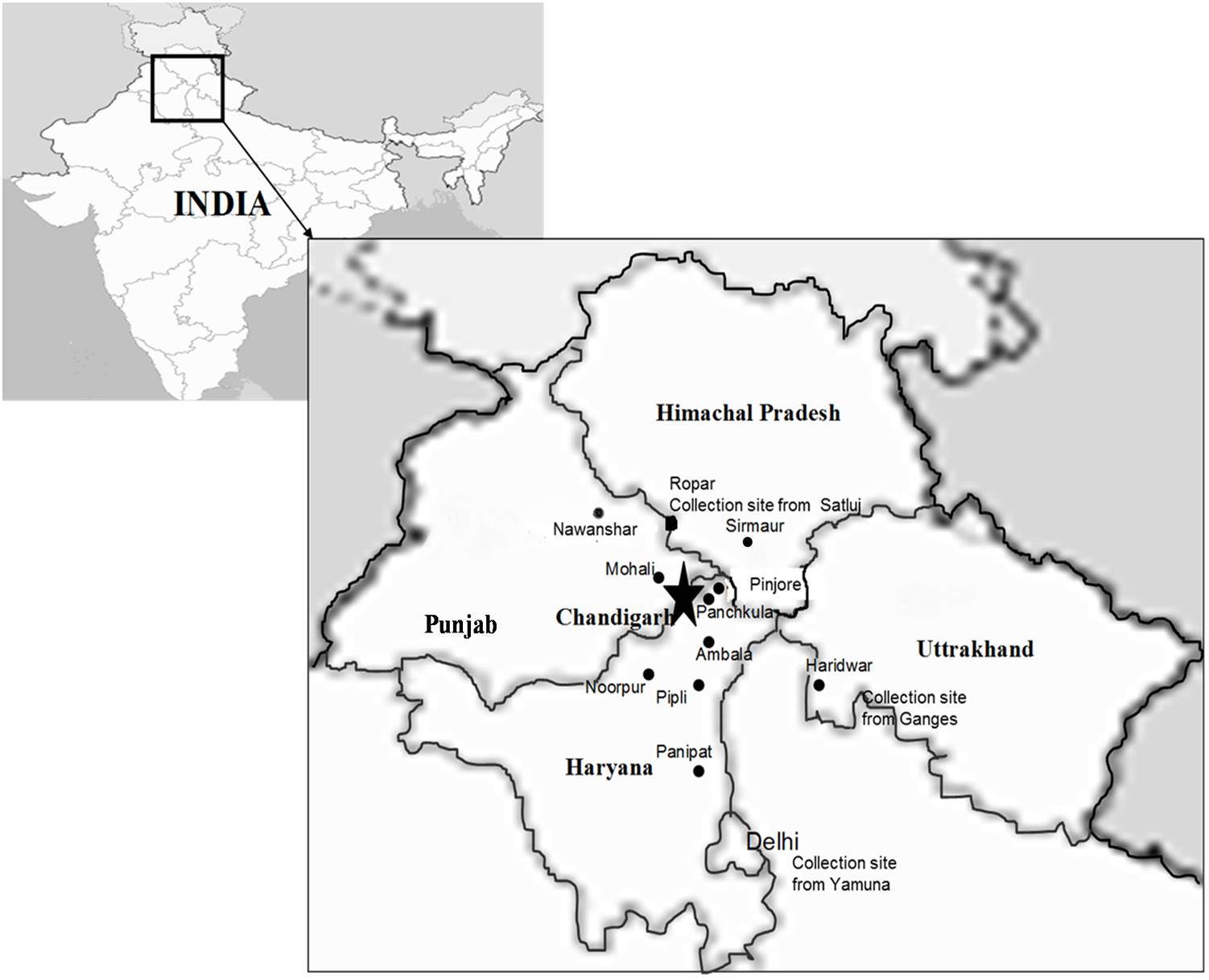

Supplement: Additional file 2 — Figure showing region of collection of clinical samples and environmental samples from random natural water sites in north India. The clinical V. cholerae O1 included in this study were collected from - Mohali, Nawanshar (Punjab), Rally (near Panchkula), Panchkula, Ambala and Noorpur (Haryana). The samples from rivers of North India viz. Satluj, Yamuna, and Ganges were collected at Bhakra (Ropar, Punjab), Delhi and Haridwar (Uttrakhand) respectively. The freshwater samples were also collected from Sirmaur (Himachal Pradesh) and Pipli (Haryana). [file 1471-2334-11-249-S2.JPEG]
