# Supplementary material for: Amplified fragment length polymorphism of clinical and environmental Vibrio cholerae from a freshwater environment in a cholera-endemic area, India
Source: BMC Infect Dis. 2011 Sep 22;11:249. doi: 10.1186/1471-2334-11-249 (PMC3206463; doi:10.1186/1471-2334-11-249)
Supplement: Additional file 3 — Table showing sequence of primers and adapters. Sequences of primers and adapters used in this study. [file 1471-2334-11-249-S3.DOC]

| **Oligonucleotide name** | **Sequence (5’ to 3’)** | **Enzyme end** |
| --- | --- | --- |
| Adapters | 5’-CTCGTAGACTGCGTACC  CATCTGACGCATGGTTAA-5’  5’-GACGATGAGTCCTGAG  TACTCAGGACTCAT-5’ | *Eco*RI  *Mse*I |
| Preamplification primers  (+0) | 5’-GACTGCGTACCAATTC-3’  5’-GATGAGTCCTGAGTAAC-3’ | *Eco*RI  *Mse*I |
| Selective primers  (+1) | 5’-GACTGCGTACCAATTCN-3’  5’-GATGAGTCCTGAGTAACN-3’  N-A/C/G/T | *Eco*RI  *Mse*I |

The primer and adapter sequences used in this study were previously described by Vos P *et al.* 1995 in *Nucleic Acid Research* 23; 4407-4414.
